# Time trend of clinical cases of Lyme disease in two hospitals in Belgium, 2000–2013

**DOI:** 10.1186/s12879-017-2841-7

**Published:** 2017-12-05

**Authors:** Mathilde De Keukeleire, Sophie O. Vanwambeke, Benoît Kabamba, Leila Belkhir, Philippe Pierre, Victor Luyasu, Annie Robert

**Affiliations:** 1 Earth and Life Institute (ELI), Georges Lemaitre Center for Earth and Climate Research, Université catholique de Louvain (UCL), Louvain-la-Neuve, Belgium; 2 Institut de Recherche Expérimentale et Clinique (IREC), Pôle Epidémiologie et Biostatistique, Faculté de Santé Publique (FSP), Université catholique de Louvain (UCL), Bruxelles, Belgium; 3 Division of Clinical Biology, Cliniques Universitaires Saint-Luc, Université catholique de Louvain (UCL), Bruxelles, Belgium; 4 Department of Internal medicine and infectious diseases, Cliniques Universitaires Saint-Luc, Université catholique de Louvain (UCL), Bruxelles, Belgium; 5 Neurology Department, Cliniques St-Pierre, Ottignies, Belgium

**Keywords:** Lyme disease, Belgium, Clinical cases, Lyme Borreliosis

## Abstract

**Background:**

As several studies indicated an increase in Lyme disease (LD), notably in neighbouring countries, concerns have arisen regarding the evolution of Lyme disease in Belgium. In order to confirm or infirm the increase of LD in Belgium, we focused on hospital admissions of patients diagnosed with LD between 2000 and 2013 based on hospital admission databases from two hospitals in Belgium.

**Methods:**

Hospital databases are a stable recording system. We did a retrospective analysis of the medical files of patients hospitalized with Lyme disease in two Belgian hospitals between 2000 and 2013.

**Results:**

The annual number of cases of LD for the two studied Belgian hospitals remained stable between 2000 and 2013, ranging from 1 for the Cliniques universitaires Saint-Luc to 15 for the the Clinique Saint-Pierre. No increasing trend were noted in the estimated annual incidence rate but the average estimated annual incidence rate was higher for the hospital Saint-Pierre (8.1 ± 3.7 per 100,000 inhabitants) than Saint-Luc (2.2 ± 1.5 per 100,000 inhabitants). The number of hospital cases of LD peaked between June and November.

**Conclusions:**

Based on hospital admissions with LD, no increasing trend was observed for the period 2000–2013 in the two studied Belgian hospitals. This is in line with other studies carried out in Belgium.

## Background

Lyme borreliosis, also called Lyme disease (LD), is the most-common tick-borne human disease in the Northern hemisphere [1, 2]. LD is a multisystemic disease caused by the spirochete *Borrelia burgdorferi* (*Bb*) which is transmitted to humans during the blood feeding of a Ixodes tick infected with *Bb* [3, 4]. The clinical manifestations of LD may vary according to the genospecies involved although all pathogenic genospecies may cause erythema migrans (EM), a typical skin lesion of LD which represent 91% of Lyme borreliosis diagnosis in the Netherlands [5–7]. In the absence of antibiotic treatment, the spirochete can disseminate and cause early disseminated LD (5.3% of diagnosis [7]) associated with Lyme neuroborreliosis, Lyme carditis, multiple EM and borrelial lymphocytoma (skin lesion) [4, 5]. Lyme arthritis can occur during this stage but it is more commonly observed during the late disseminated stage [4]. Late LD is also regularly associated with skin disorder known as acrodermatitis chronica atrophicans and rare neurological manifestations [4, 5]. LD diagnostic is mainly based on clinical symptoms (presence of EM, facial palsy, arthritis), clinical history, and on the demonstration of a serological response to *Bb*, except for the early stages [8].

Over the last decades, LD has become a hot topic in Europe and North America as several studies indicated an increase in the incidence of this disease, e.g. in The Netherlands and in Germany (eastern states), whereas others reported no trend as in Switzerland [1, 2, 9–13]. Given that the incidence of LD may be increasing in neighbouring countries, concerns have arisen regarding the evolution of LD in Belgium. Since LD is not a notifiable disease in Belgium [2], knowledge is up to now mainly based on epidemiological surveillance. Two studies investigated the incidence of the disease in Belgium based on data from a sentinel network of general practitioners [14], or from the registration of minimal clinical (RMC) data from Belgian hospitals and from a sentinel laboratory network reporting positive laboratory results [15]. The results of those two studies showed no increase in LD over time for the 2003–2010/2012 period in Belgium. Results based on these two sources of data can be challenged because they are based on voluntary reporting from general practitioners and laboratory participating to these networks, possibly resulting in underreporting, or uneven reporting, of cases. Medical staff can be more or less active in reporting cases, depending on their awareness of the subject. This may induce spatial and temporal variability in the data reported. Moreover, a RMC is a systematic report of hospital cases but it does not allow to identify patients and it is therefore impossible to distinguish several patients from several hospitalisations for a single patient; incidence can therefore be overestimated.

In order to monitor the evolution over time of LD in Belgium and to assess time trends in LD in Belgium, we conducted a study on clinical series of patients diagnosed with LD in two hospitals in Belgium.

## Methods

### Data extraction

All patients hospitalized with LD between 1/01/2000 and the 31/12/2013 were retrieved from medical records of two hospitals in Belgium: the Cliniques universitaires Saint-Luc (CUSL) and the Clinique Saint-Pierre (CSPO) in Ottignies-Louvain-la-Neuve. CUSL is located in the eastern part of the urban nucleus of Brussels, with 979 certified beds out of the 5691 (17%) for the Region Bruxelles-Capitale (Fig. 3) and it is a top-level hospital in the Belgian health system [16]. CSPO is a regional hospital situated at the heart of the province of Walloon Brabant (Fig. 3), mostly rural area, with 425 certified beds out of the 1618 (26%) for the Walloon Brabant, and it is a first-level teaching hospital in the Belgian health system [16].

Since 1990, all Belgian hospitals have to report clinical registered data (registration of minimal clinical data, RMC, also called minimal hospital summary (RHM) since 2008) to the Federal Public Health Service [17]. The RHM is issued to the autoritities mainly for hospital financing purposes. The record is a standardized and concise summary of medical files that physicians have to fill for each patient. All diagnoses are recorded as well as some patient data, length of stay, treatment. Data are summarized and then coded following the International Classification of Diseases-9 (ICD-9). In the ICD-9 classification, all Lyme borreliosis diseases are grouped under the code 088.81. The first step in the data extraction process was to screen the RHM databases of the two hospitals for the selected consecutive 14 years study period and export all files where a code 088.81 appeared. To avoid missing any cases of LD, we also extracted files countaining codes directely associated to LD: EM, borrelial lymphocytoma, acrodermititis chronica atrophicans, meningitis, radiculoneuritis, meningo-radiculoneuritis, bannwarth syndrome, polyneuropathy, atrioventricular block, carditis, oligoarticular arthritis, arthritis, cranial nerve, meningoencephalitis, encephalomyelitis, cerebral vasculitis, myositis, eye (conjunctivitis with keratitis with endophtalmitis with panophthalmitis). The second step was to analyse medical files for every patient selected in the first step. Medical files were reviewed by two physicians who checked if the patient was diagnosed as a LD, and a third one in case of discrepency.

The study protocol was approved by the Ethics Committee of Université catholique de Louvain Medical School, Belgian Registry N° B40320096360 and by the Ethics Committee 045 of Clinique Saint-Pierre in Ottignies.

### Data analysis

Hospital admissions for LD from CUSL and CSPO were analysed regarding epidemiological aspects (age, sex, seasonality) and clinical aspects. The geographical distribution was examined based on the place of residence.

The annual estimated incidence rate of hospitalisations with LD in those two hospitals was calculated as follows: the number of hospitalisations in each hospital per year was divided by the population size in the province each year and multiplied by the importance of the hospital in that geographical region. The importance of the hospital was measured by the ratio between the number of certified beds in the hospital (979 for CUSL and 425 for CSPO) and the total number of certified beds in the considered geographical region.

## Results

From 2000 to 2013, 57 hospital admissions with a final diagnosis of LD were recorded in CUSL and 110 in CSPO.

### Epidemiological aspect

**Fig. 1:**
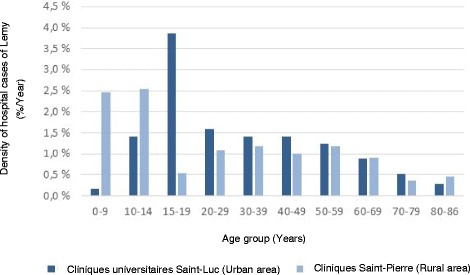
Density of hospital cases of Lyme disease by age group in two hospitals of Belgium (2000–2013)

Hospital admissions were mainly women in CSPO (62%) and men in CUSL (61%) (Table 1). The youngest patient was 2 years old and was admitted in CSPO for neuroborreliosis, like the oldest patient (86 years old). The age distribution for CSPO showed a peak for young people (0–9 years) whereas the age distribution for CUSL showed a peak for people aged of 15–19 years (Fig. 1). 28% of admitted cases in CUSL and 40% in CSPO were aged under 20 years.

**Table 1:** Socio-demographic characteristics of patients hospitalized with LD in two hospitals of Belgium (2000–2013)

		Cliniques universitaires Saint-Luc (Urban area)			Clinique Saint-Pierre (Rural area)		
		n	Mean ± SD	%	n	Mean ± SD	%
Age		57	36.3 ± 19.9		110	32.4 ± 23.3	
	0–9	1		1.8	27		24.5
	10–14	4		7.0	14		12.7
	15–19	11		19.3	3		2.7
	20–29	9		15.8	12		10.9
	30–39	8		14.0	13		11.8
	40–49	8		14.0	11		10.0
	50–59	7		12.3	13		11.8
	60–69	5		8.8	10		9.1
	70–79	3		5.3	4		3.6
	80 et plus	1		1.8	3		2.7
Sexe		57			110		
	Women	22		38.6	68		61.8
	Men	35		61.4	42		38.2

**Fig. 2:**
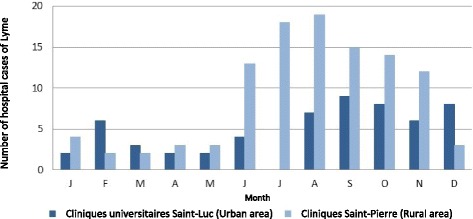
Number of hospital cases of Lyme disease by months (2000–2013) in two Belgian hospitals

The number of hospital admissions (per month) with Lyme disease during the period 2000–2013 follows a seasonal pattern in the two hospitals (Fig. 2). This number peaked between June and November: 60% and 84% of admissions in CUSL and in CSPO were recorded between June and November, respectively.

**Fig. 3:**
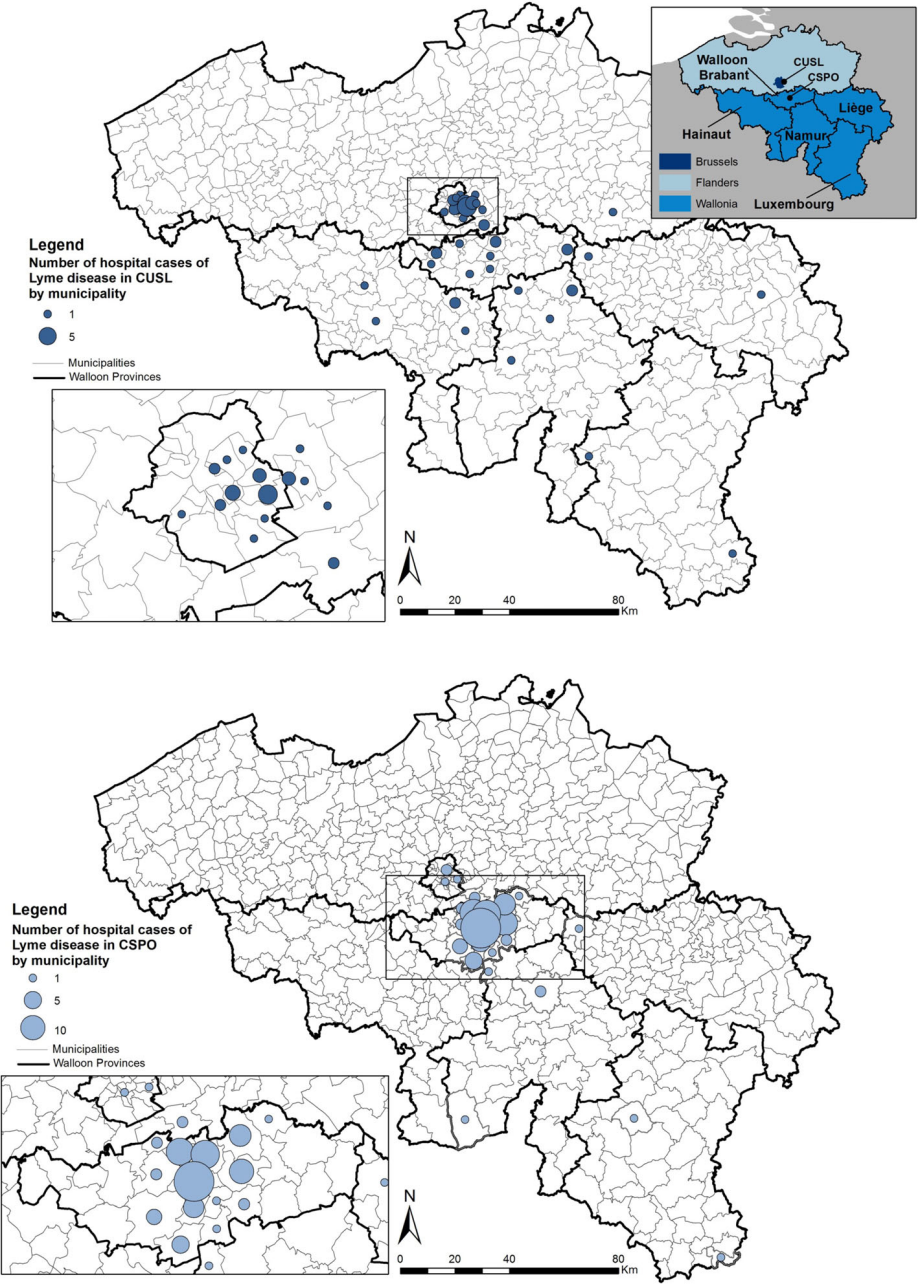
Spatial distribution of hospital cases of Lyme disease (2000–2013) in two hospitals of Belgium according to the patient’s residence municipality

Patients who were diagnosed for Lyme disease were coming from 51 different Belgian municipalities (Fig. 3). Patients admitted to CUSL were mainly coming from Brussels (39%) but also from various Walloon and some Flemish municipalities. 64% of patients who were admitted to CSPO were coming from the province of Brabant Walloon, and more precisely, 56% were coming from municipalities close to the hospital: Ottignies-Louvain-la-Neuve, Wavre, Rixensart and Chaumont-Gistoux.

### Clinical aspect

**Fig. 4:**
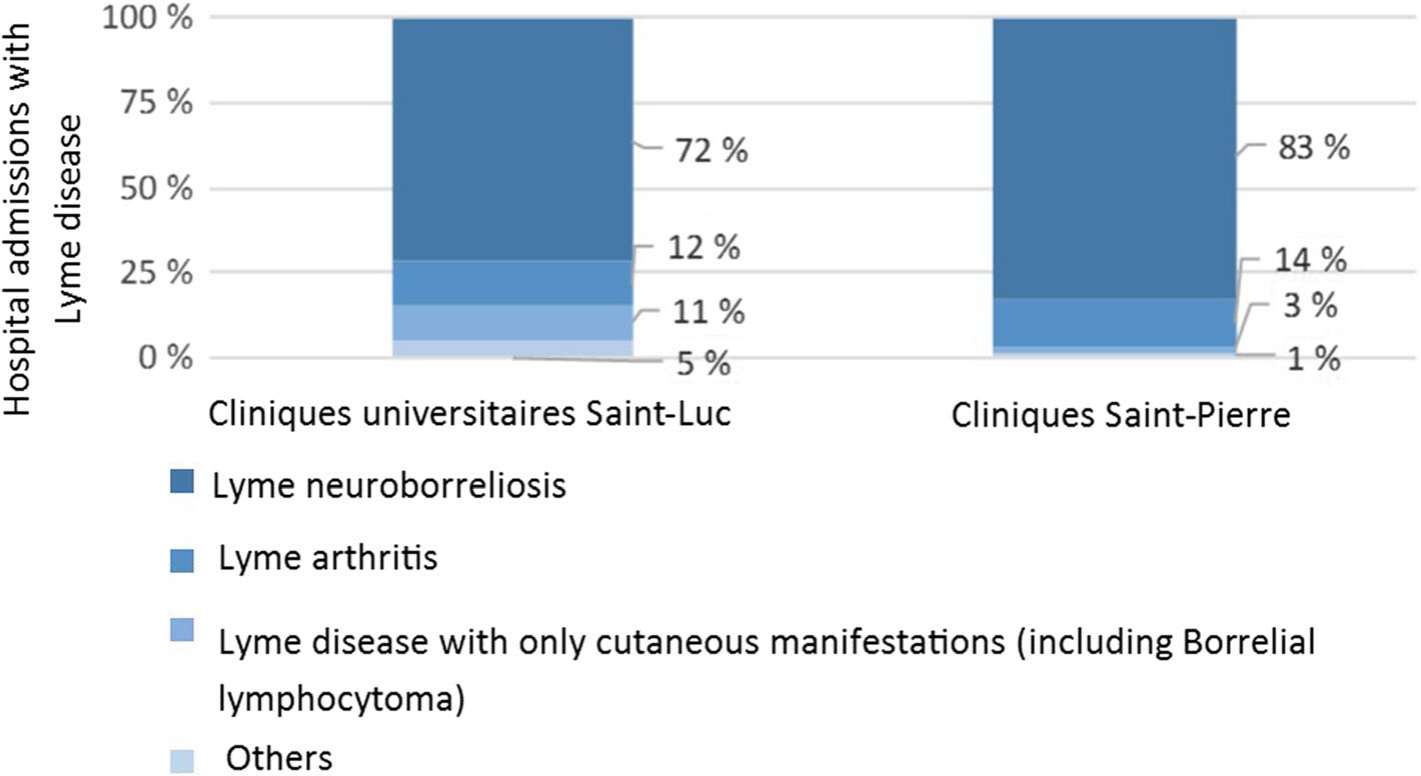
Hospital cases of Lyme disease according to clinical diagnosis in two Belgian hospitals (2000–2013)

The main clinical diagnosis was neuroborreliosis in the two hospitals: 72% in CUSL and 83% in CSPO (Fig. 4). Cutaneous manifestations were present at the time of hospital admission for 11% in CUSL and 3% in CSPO. Diagnose of Lyme arthritis were equivalent in the two hospitals. Others were panuveitis (1 case in CUSL), uveitis (1 case in CUSL) or carditis (1 case in CUSL and 1 case in CSPO).

**Fig. 5:**
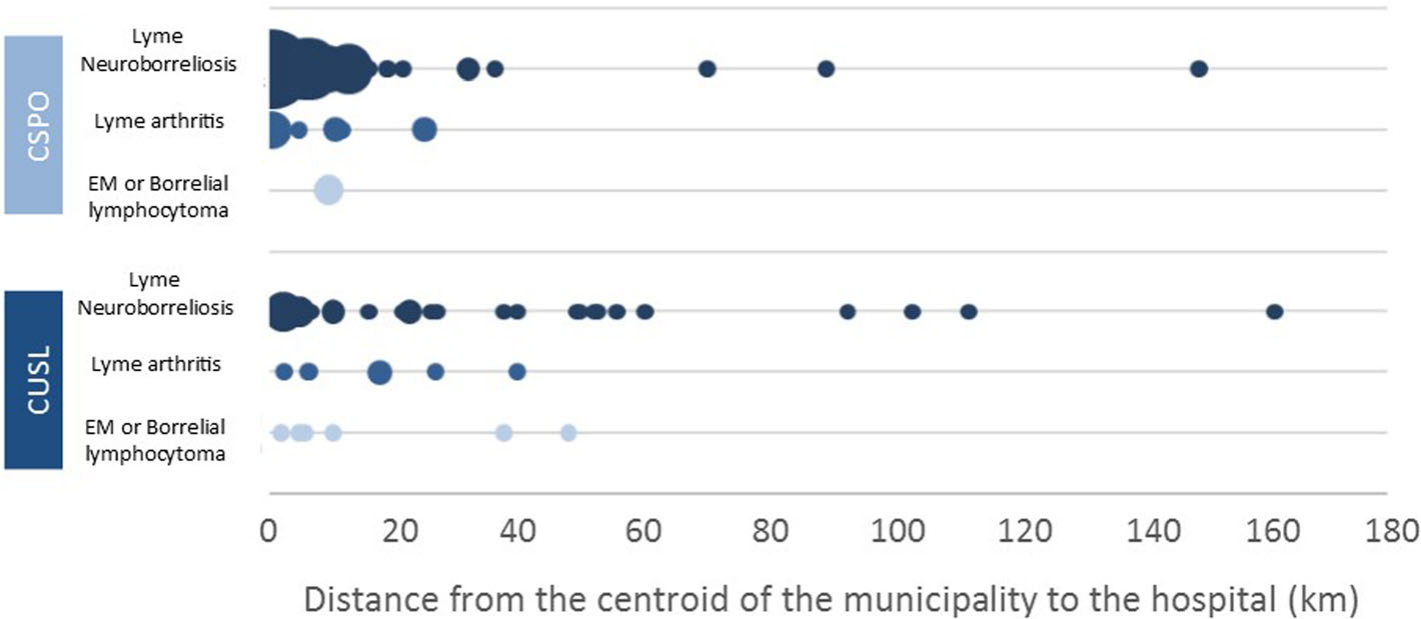
Clinical diagnosis of hospital cases of Lyme disease in two Belgian hospitals (2000–2013) according to the distance to the hospital (Bubble size is propotionnal to the number of cases per municipality)

People diagnosed with cutaneous manifestations (EM or a Borrelial lymphocytoma) travelled on average 15 ± 8 km from their residential municipalities to the hospital (Fig. 5): the three cases hospitalized in CSPO for cutaneous manifestations were from neighbouring municipalities of the hospital and, in CUSL, four among the six cases hospitalized for aspecific symptoms with also cutaneous manifestations were from Brussels municipalities, and their hospital stay was less than 2 days. People diagnosed with Lyme neuroborreliosis, a more severe form of LD, were on the contrary coming from more distant municipalities and some travalled more than 100 km.

**Fig. 6:**
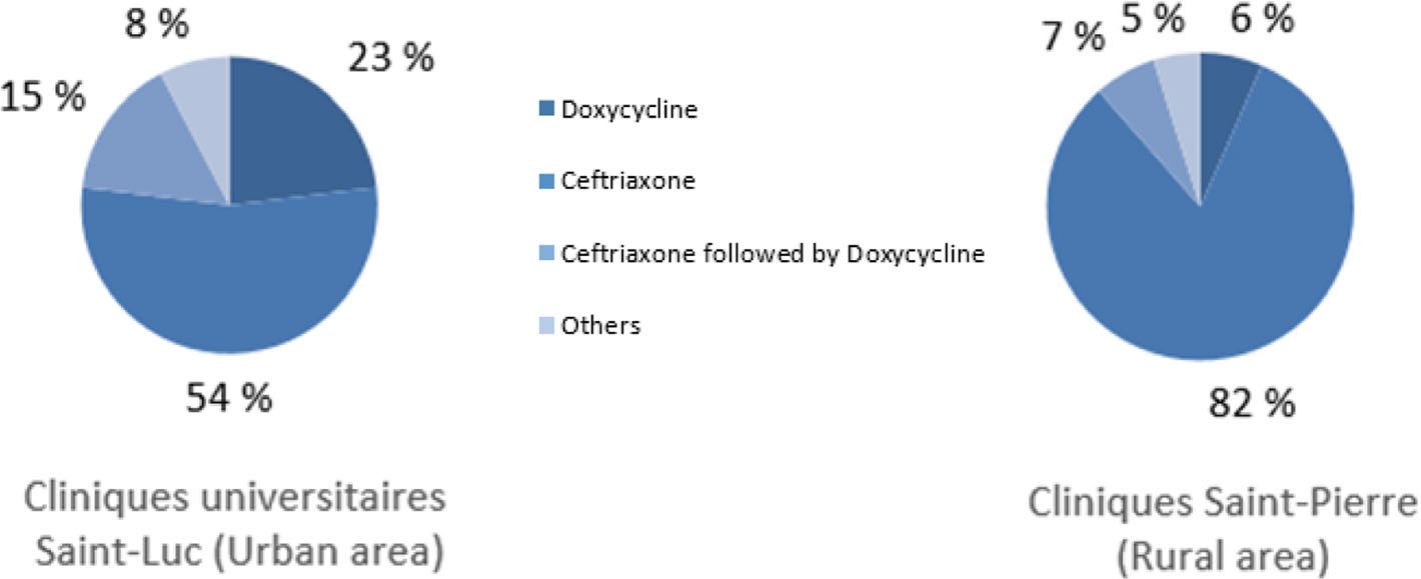
Treatment of hospital cases of Lyme disease in two Belgian hospitals (2000–2013)

The average stay duration was similar in the two hospitals: 5.5 ± 4.0 days in CUSL and 5.6 ± 3.0 days in CSPO. Patients were treated with ceftriaxone (53.8% in CUSL and 82.0% in CSPO), doxycycline (23.1% in CUSL and 6.6% in CSPO) or a combinaison of both, firstly ceftriaxone and then doxycycline (15.4% in CUSL and 6.6% in CSPO) but doxycycline was more frequently prescribed in recent years (Fig. 6).

### Annual estimated incidence rate of hospitalisations

**Fig. 7:**
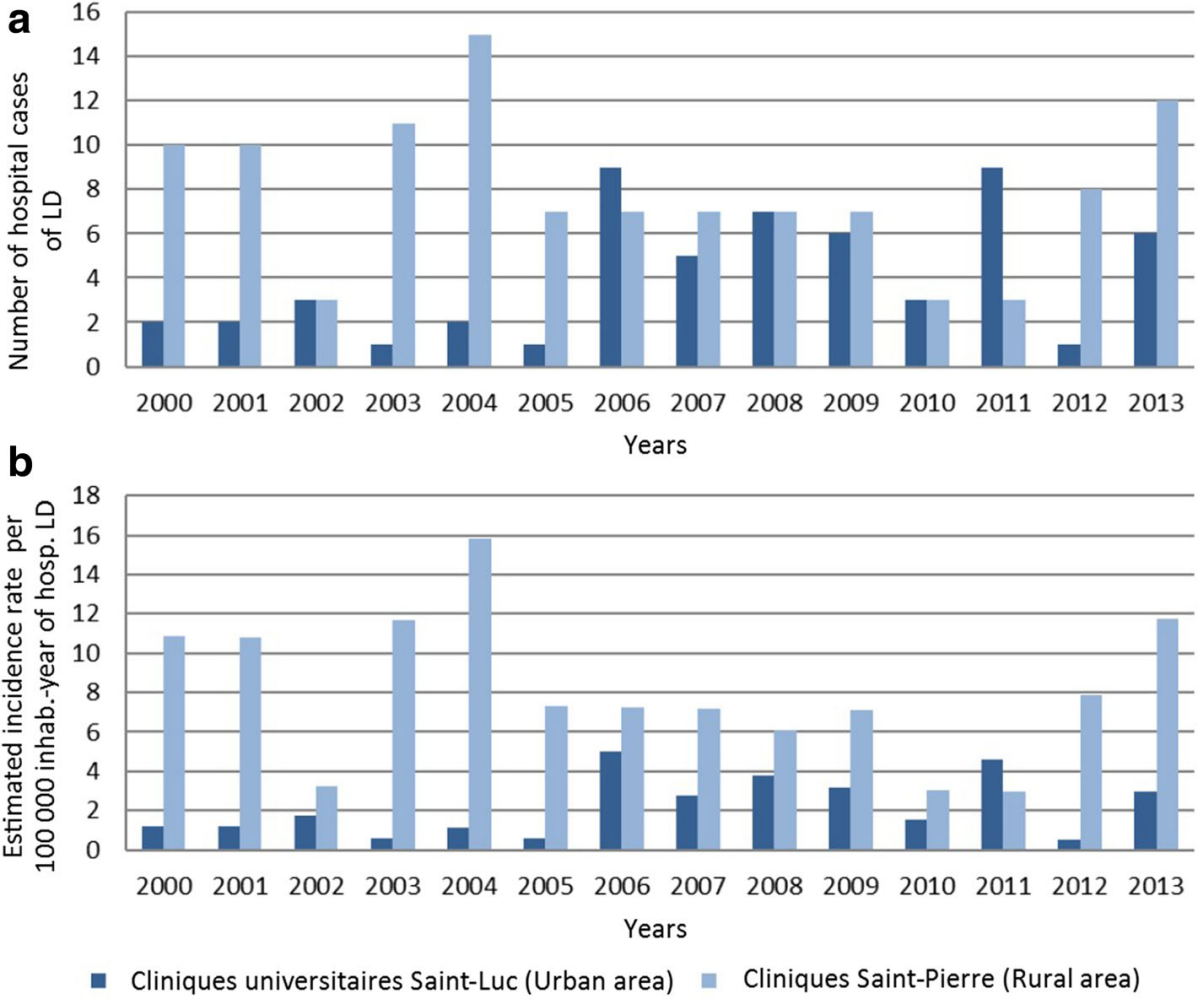
Annual number of hospital cases of Lyme disease (**a**) and annual estimated incidence of hospital cases of Lyme disease per 100,000 inhabitants (**b**) in two hospitals of Belgium between 2000 and 2013

Figure 7a presents the number of hospitalisations with LD per year for the two hospitals. No increasing trend was observed during the study period for the CUSL but a jump was noticed in 2006: 2 ± 1 cases per year for the period 2000–2005 (−0.20 ± 0.17,*p*-value = 0.32) and 6 ± 3 cases per year for 2006–2013 (−0.45 ± 0.32, *p*-value = 0.33) with a maximum of 9 cases in 2006 and 2011. A mean of 9 ± 4 cases per year was observed for CSPO for the period 2000–2005 and 7 ± 3 cases per year for 2006–2013, without any increasing time trend (slope of −0.20 ± 0.24, *p*-value = 0.41). The number of admissions was 3 for 2002, 2010 and 2011 and the maximum was observed in 2004, with 15 cases.

The annual estimated incidence rate of hospital cases of LD were calculated for the two hospitals (Fig. 7b). For CUSL, the average annual estimated incidence rate of hospital cases of LD over the period 2000–2013 was 2.2 ± 1.5 per 100,000 inhabitants, with a minimum of 0.50 in 2012 and a maximum of 5.03 in 2006. The average annual estimated incidence rate of hospital cases over the period 2000–2013 was 8.1 ± 3.7 per 100,000 inhabitants for CSPO, ranging from 2.98 in 2011 to 15.83 in 2004. Over the period 2000–2013, the cumulative estimated incidence of hospitalizations with LD was 34 and 119/100000 inhabitant-years for CUSL (urban area) and CSPO (rural area), respectively (Table 2).

**Table 2:** Trend slopes calculated for the number of hospitalisations and the annual estimated incidence rate of hospitalisations in two hospitals of Belgium (2000–2013)

	Cliniques universitaires Saint-Luc		Clinique Saint-Pierre
	2000–2005	2006–2013	2000–2013
Number of hospitalisations	−0.20 ± 0.17	−0.45 ± 0.32	−0.20 ± 0.24
Annual estimated incidence rate of hospitalisations	−0.13 ± 0.10	−0.30 ± 0.22	−0.28 ± 0.24

Because trend slopes calculated for the number of hospitalisations and the annual estimated incidence rate of hospitalisations are comparable, conclusions drawing from numbers are similar to conclusions drawing from estimated incidence rates of hospitalisations.

To have an idea of how many cases it can be represent for Belgium, we can extrapolate our data knowing that CUSL represent 1.4% of certified Belgian beds and CSPO 0.6%. As a total of 12 cases were on average recorded per year in the two hospital, we can reach a total of 600 hospitalized cases of LD per year for Belgium.

## Discussion

Lyme borreliosis has become a hot topic in Europe and North America as several studies indicated an increase in this disease whereas others reported no trend. In the present study, the annual number and the annual estimated incidence rate of hospital cases of LD did not show any increase between 2000 and 2013 in the two Belgian hospitals. As expected, the average annual estimated incidence rate was higher in CSPO, that is located in a rural area, (8.1 ± 3.7 cases per 100,000 inhabitants per year) than CUSL, that is located in an urban area (2.2 ± 1.5 cases per 100,000 inhabitants per year). As in most studies, the number of hospital admissions followed a seasonal pattern. In the two hospitals, the main clinical diagnosis was neuroborreliosis, the most frequent severe form of the disease.

This study is one of the first analyzing hospital cases of LD during 14 consecutive years in Belgium. It gives a view of the time trends of LD in hospitals during this period. Two previous studies have been conducted in Belgium based on data from the sentinel network of general practitioners, the sentinel laboratory network and the registration of minimal clinical (RMC) data from Belgian hospitals [14, 15]. The sources of data in these two studies were based on voluntary reporting from general practitioners and laboratory participating to these networks, possibly resulting in an underreporting of cases. In RMC database, there is no difference between one patient hospitalized two times and two patients hospitaled one time. Contrary to these three sources of data, every patient diagnosed with LD in the two studied hospitals was retrieved and we are able to distinguish LD diagnostic from a simple request for Lyme investigation. Our methodology allowed us to attest that every patient was counted only once. Besides, as a hospital database is a stable system, bias are unlikely and hospital records provide therefore useful data for trend analyses. Despite suspected biases with these data, the results reported by these approaches are in line with those stated in this study: i.e. a notable absence of a disease increase in the population.

Our study has some limitations. Firstly, mistakes may be present in the database issued for data extraction (RHM), in relation to the diagnosis, data entry, or unintentional ommissions. Secondly, hospital admissions only give us a partial idea of the incidence of LD in Belgium because very few patients presenting Lyme disease need to be hospitalized. For example, people suffering from only an EM are generally consulting a general practitioner instead of hospitals. However, another study using data of the Belgian network of sentinel general practitioners reassure our conclusion: estimated incidence rate of EM did not increase between 2003/2004 and 2008/2009 [14]. Thirdly, two hospitals only were considered; other hospitals may have other results. Fourthly, as people do not necessarily remember being bitten, or where, medical files do not always specify the place of infection and residence is thus the sole option for mapping patients.

However, the use of hospital databases is a powerful and stable tool to study LD to monitor trends, to study seasonality, to assess incidence, and to provide characteristics of hospitalized patients.

Concerning the age of patients suffering from LD, a peak at young age was noticed. This is consitant with many studies reporting that children and elderly people are at a higher risk [2, 12, 14, 15, 18]. Our results showed a marked difference in the sex ratio between the two hospitals: hopital admissions concerned women in 39% of cases in the hospital located in an urban area and 62% in the one located in a rural area. Within the last, women were mainly 18 to 64 years old (32% of the population) and men were mainly 18 to 64 years old in the second hospital (42% of the population). No consensus appears regarding this subjet [2]. In Belgium, higher LD incidence in men than in women has previously been described [14, 15] but the contrary too [3]. The influence of gender on the risk of contracting tick bites was reported but no conclusions about factors causing those differences (biologic, immunologic, or sociological mechanisms) have been made by Authors [19].

The number of hospital admissions by month of entry for LD follows a seasonal pattern: fewer admissions in winter, an increase during spring and summer with a peak at the end of summer and gradual decrease during autumn. This seasonal pattern was also found in other studies in Belgium [3, 15] as well as in neighbouring countries [1, 20, 21]. Human risk of contracting LD, and more generally tick-borne diseases, is directly linked to the frequence of contacts between humans and infected ticks [22]. Prevailing factors are the phenology and the distribution of ticks which are affected by environmental conditions. In Belgium, the abundance of ticks starts to increase in spring, reach a peak in summer and decrease in automn [23]. Superimposed on those factors is human behaviour: humans are more exposed to tick bites when visiting tick habitats, or less exposed by using prevention or protection measures. In Belgium, people are more likely to prefer spring and summer to visit forests and are so more exposed [24]. Therefore, the highest risk of LD arises when tick activities peak and humans visit tick infested areas [10]. The monthly distribution of hospital cases, most numerous in the months following summer, is coherent with the seasonality of highest risk.

Admissions with LD were recorded for people living in urban municipalities in both hospitals. Because the spatial distribution of patients is based on the place of residence, which does not always reflect the place of infection, these people were either bitten in other municipalities where they entered tick habitats, or there are ticks in urban parks.

Because distance has friction, hospitals, as other falicilites, show a drop in use with greater distance to overcome [25]. The distance that people travel to go to the hospital and so the geographic distribution of patients varies with the hospital level. In the rural hospital which is a first level hospital in the Belgian healthcare structure, people are mostly coming from nearby municipalities whereas in the second hospital, an university hospital, people are coming also from various municipalities situated at farther distances. The clinical signs are related to the travelled distance between the residence municipality and the hospital: people suffering from more severe forms of LD, such as neuroborreliosis, are willing to travel longer distances to come to a high level hospital than people suffering from less severe forms such as EM. It should be underlined that few EM were still visible at the time of admission in both hospitals, reflecting the fact that people are generally consulting a general practitioner for EM. More EM at admission were recorded in CUSL than in CSPO but concerned patients were living close to the hospital. It can also be pointed out that very severe manifestations such as panuveitis, uveitis and carditis were more frequently reported in the university hospital (3 cases) than in CSPO (1 case).

The main clinical form was neuroborreliosis followed by Lyme arthritis and cutaneous manifestations. This distribution is in line with the species of Borrelia observed in Belgium in a study conducted in 1998 [26]. Prevalent genospecies in Belgian ticks were *B. garinii* (53% of infected ticks), *B. burgdorferi ss* (38%) and *B. afzelii* (9%). Those results were updated in 2014 and the dominance of *B. garinii* (54% of infected ticks) was confirmed, followed by *B. valaisiana* (27%), *B. burgdorferi ss,* and *B. afzelii* (9%) (Badalamenti, J: Prévalence et diversité génétique de Borrelia burgdorferi sensu lato chez Ixodes ricinus et étude des facteurs environnementaux influençant sa distribution en Belgique, unpublished). *B. garinii* seems to be the most neurotropic genospecies, the main observed sign in our study, whereas *B. burgdorferi ss* seems to be the most arthritogenic. *B. afzelli* is mostly associated with skin manifestations [5, 6], which are less observed in our study.

The annual estimated incidence rate of hospitalisations with LD in those two hospitals was calculated as the result of the number of hospitalisations in each hospital per year divided by the population size in the province each year and multiplied by the importance of the hospital in that geographical region measured using certified beds. Using this method, we made different implicit assumptions. First, we assumed that use of hospitalisations for LD is constant over time, which seems realistic. Secondly, we hypothesize that the geographical coverage of hospitals is constant over time. Indeed, attractiveness of the two hospitals did not change between 2000 and 2013. In Saint-Pierre, a general hospital located in the eastern part of the province of Walloon Brabant, people are mostly coming from nearby municipalities. Given that other general hospitals are located in the western part of the province, the attractiveness of Saint-Pierre is quite stable. The attractiveness of Saint-Luc is also constant because it is a first-level teaching hospital in the Belgian health system. Thirdly, in our calculation, the importance of hospital depends on the percentage of certified beds that remains constant during the period 2000–2013. It is also important to note that analyzing annual estimated incidence rates of hospitalisations we saw trend comparable to those observed for the number of hospitalisations.

The average annual estimated incidence rate in the rural hospital was 8.1 ± 3.7 per 100,000 inhabitants per year and it was 2.2 ± 1.5 per 100,000 inhabitants per year in the urban hospital, for the period 2000–2013. Similar incidence values were reported in neighboring countries. An estimated annual average hospitalisation rate of 1.55 per 100,000 inhabitants in France (2004–2009) with rates ranging from 2.1 to 4 in the North-East [1]. In Germany also, the mean nationwide inpatient incidence was 9/100000 over the period 2008–2011 [27].

Our results showed that the annual number as well as the annual estimated incidence rate of hospital admissions with Lyme between 2000 and 2013 was higher in CSPO than in CUSL. This can be explained by the location and the status of the two hospitals. CSPO is surrounded by rural areas where people are at a higher risk of tick bites than people living in urban areas as the area covered by CUSL. Moreover, CSPO is a first level hospital in the healthcare structure in Belgium whereas CUSL is an university hospital. As the management of LD does not require specialised technologies, it can be implemented in any healthcare structure, as far the western blot test is available for confirming the diagnosis of LD. However, resorting to the use of a first level hospital is generally more common for reasons of shorter waiting times or distances.

The results of this study showed that there is no increasing trend in the estimated incidence rate of hospital cases of LD between 2000 and 2013. This corroborated the results of those two studies which showed no increasing trend in LD during 2003–2010/2012 period in Belgium [14, 15]. Although some neighbouring countries found an increasing trend in the incidence of this disease, e.g. in The Netherlands, but not confirmed in their more recent paper [7], or Germany (eastern states) [12], others reported no marked trends. In France, the number of hospitalised cases remained stable for the period 2004–2009 [1]. The same was observed in Switzerland between 2008 and 2011 [21]. Comparaisons must be done cautiously as surveillance methodologies differ between countries. Other factors can also explain variations: awareness of the disease, serological tests used, reimbursments of tests, etc.

Although our results suggest that the estimated incidence rate of hospital cases of LD did not increase during the period of the study, it should be pointed out that such findings do not allow to conclude that LD is not increasing in Belgium. Indeed, currently, many LD cases are managed by general practitioners, without any hospitalization, and even often without any serological test when LD signs are evident. So, assessing today the actual number of LD cases in Belgium is quite impossible.

Our study has sereval implications. In terms of research, databases of hospital admissions with LD are a good tracer to study the temporal dynamic of LD. Various methods exist to assess LD risk but one way to asses the importance of LD is to analyse human cases. In Belgium, since LD is not notifiable, four systems are used to collect data about LD [28]: reporting from two laboratories constituting the national reference centers for LD, from a sentinel network of general practitioners, from a sentinel laboratory network, and from the registration of minimal clinical (RMC) data from Belgian hospitals. However, as the report is not systematic, the results can be influenced by various factors external to the actual variation in LD, like the awareness of medical staff to the disease. However, the fact that our results confirmed the conclusion of two other studies [14, 15] indicates that those epidemiological datasets displayed well the trend of the disease in Belgium. Hospital databases are a stable sytem and biases are unlikely beside other data as sentinel network of general practitioners or as sentinel laboratory network which can be influenced over time (awareness of the disease, serological tests used, etc.). Moreover, although reviewing all medical files is tedious, all patients diagnosed with LD are taken into account and so the situation of LD in hospitals is more precisely known. In terms of public health, our study confirms once again that LD is endemic in Belgium. Public health measures should be maintained to inform people about the risk of tick bites and about the ways to prevent tick bites, in order to reduce the risk of LD.

## Conclusion

In conclusion, this study is the first to estimate the epidemiology of LD in Belgium based on hospital admission databases. As other previous studies conducted in Belgium, our study shows that, between 2000 and 2013, there is no increasing trend in the annual estimated incidence rate of hospital cases of LD in the two studied hospitals. Our results are in line with other recent studies carried out in Europe. Moreover, our study adds new information and extends the available knowledge by providing more details about clinical epidemiology of LD in Belgium.

## Abbreviations

Bb: Borrelia burgdorferi
CSPO: Clinique Saint-Pierre
CUSL: Cliniques universitaires Saint-Luc
EM: erythema migrans
ICD-9: International Classification of Diseases-9
LD: Lyme disease
RHM: Minimal hospital summary
RMC: Registration of minimal clinical
